# Supplementary material for: Starvation-induced proteasome assemblies in the nucleus link amino acid supply to apoptosis
Source: Nat Commun. 2021 Nov 30;12:6984. doi: 10.1038/s41467-021-27306-4 (PMC8633328; doi:10.1038/s41467-021-27306-4)
Supplement: Supplementary file 1 — Supplementary Information [file 41467_2021_27306_MOESM1_ESM.pdf]

**Starvation-induced proteasome assemblies in the nucleus links amino acid supply to apoptosis**

Maxime Uriarte, Nadine Sen Nkwe, Roch Tremblay, Oumaima Ahmed, Clémence Messmer, Nazar Mashtalir, Haithem Barbour, Louis Masclef, Marion Voide, Claire Viallard, Salima Daou, Djaileb Abdelhadi, Daryl Ronato, Mohammadjavad Paydar, Anaïs Darracq, Karine Boulay, Nicolas Desjardins-Lecavalier, Przemyslaw Sapieha, Jean-Yves Masson, Mikhail Sergeev, Benjamin H Kwok, Laura Hulea, Frédérick A. Mallette, Eric Milot, Bruno Larrivée, Hugo Wurtele and El Bachir Affar

**Supplementary Figures**

S1: HBSS treatment does not induce cell death at the time of proteasome foci formation.

S2: Intact proteasome particle is assembled in nuclear foci following nutrient starvation.

S3: SIPAN are not associated with nucleolar stress.

S4: SIPAN are observed in multiple cell lines and certain cancer cells have reduced ability to form SIPAN.

S5: Rapid dissipation of SIPAN after nutrient replenishment.

S6: SIPAN are highly responsive to physicochemical perturbations of the cell environment.

S7: SIPAN colocalize with conjugated ubiquitin, but not with SUMO.

S8: Liquid-Liquid Phase separation of RAD23B in vitro.

S9: Non-Essential Amino Acids (NEAA) inhibit SIPAN formation and protect from cell death that results from nutrient depletion.

S10: Inhibition of RAD23B or PSME3 promotes cell survival in response to amino acid deprivation.

S11: Example of gating.

**Supplementary Tables**

Supplementary Table 1: List of compounds

Supplementary Table 2: List of siRNA

Supplementary Table 3: List of antibodies

Supplementary Table 4: List of primers

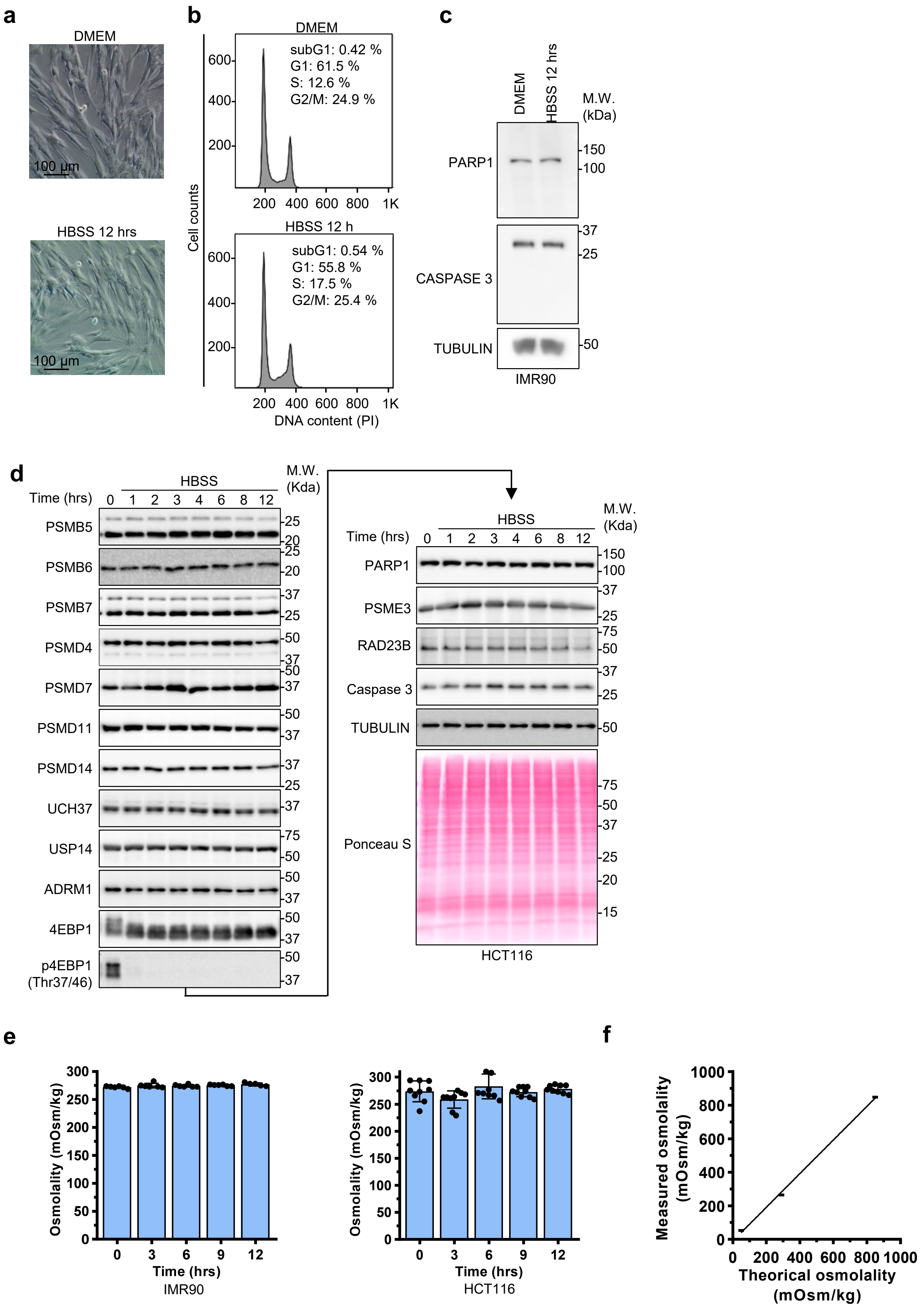


**Supplementary Figure 1**

**Supplementary Figure 1. HBSS treatment does not induce cell death at the time of proteasome foci formation. a**) Phase contrast images of IMR90 cells following incubation in HBSS for 12 hrs (Representative from 3 independent experiments). **b**) FACS analysis of IMR90 cells collected following nutrient deprivation for 12 hrs (Representative from 2 independent experiments). **c**) Protein levels of PARP1 and CASPASE-3 following nutrient deprivation in IMR90 cells for 12 hrs . Cells were incubated in HBSS solution and harvested for immunoblotting (Representative from 3 independent experiments). **d**) Protein levels of proteasome components and other factors following nutrient deprivation in HCT116 cells. Cells were incubated in HBSS solution and harvested at different times points for immunoblotting. 4EBP1 phosphorylation is included as a control for nutrient starvation (Representative from 2 independent experiments). **e**) Incubation of IMR90 or HCT116 cells in HBSS is not associated with hyperosmotic stress. IMR90 or HCT116 cells were incubated in HBSS and osmolality was measured at the indicated time points (n=3 independent experiments). **f**) Representative calibration graph conducted during measurement of osmolality (Representative from 3 independent experiments). Data in **e** and **f** the mean +/- SD (n=3 independent experiments). Source data are provided as a Source Data file.

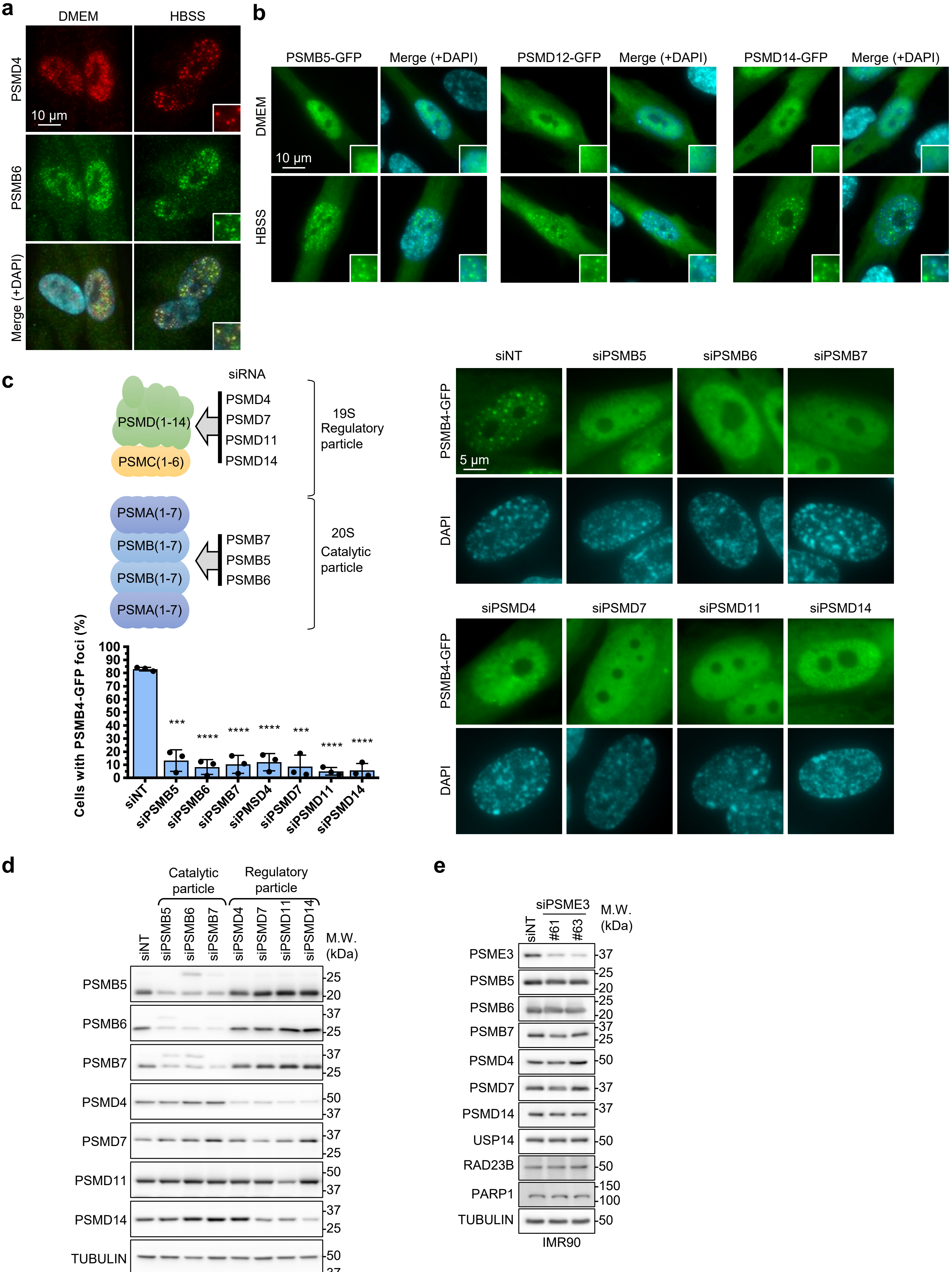


**Supplementary Figure 2**

**Supplementary Figure 2. Intact proteasome particle is assembled in nuclear foci following nutrient starvation**. **a)** PSMD4 (RP) and PSMB6 (CP) co-localize in nuclear foci in IMR90 cells after 6 hrs of nutrient deprivation (Representative from 3 independent experiments). **b)** PSMB5-GFP, PSMD12-GFP, PSMD14-GFP localize in nuclear foci following 6 hrs incubation of IMR90 cells in HBSS (Representative from 3 independent experiments). **c)** Left top, Schematic representation of proteasome components depleted by siRNAs. Right, Representative images of IMR90 cells expressing PSMB4-GFP that were depleted of proteasome components and treated with HBSS for 6 hrs. Left bottom, counts of cells with PSMB4-GFP foci are represented (n=3 independent experiments). **d)** Following siRNA-mediated depletion of components of the RP or CP, IMR90 cells were harvested after three days for immunoblotting (n=1 experiment). **e)** Depletion of PSME3 does not result in changes in the levels of proteasome proteins. Following siRNA depletion of PSME3, IMR90 cells were harvested for western blotting (n=3 independent experiments). Data represent the mean +/- SD. Cells with more than 10 foci are counted (**c**). *P<0.05; **P<0.01; ***P<0.001; ****P<0.0001; ns: not significant; 2-sided unpaired Student's t-test used in **c**. Source data are provided as a Source Data file.

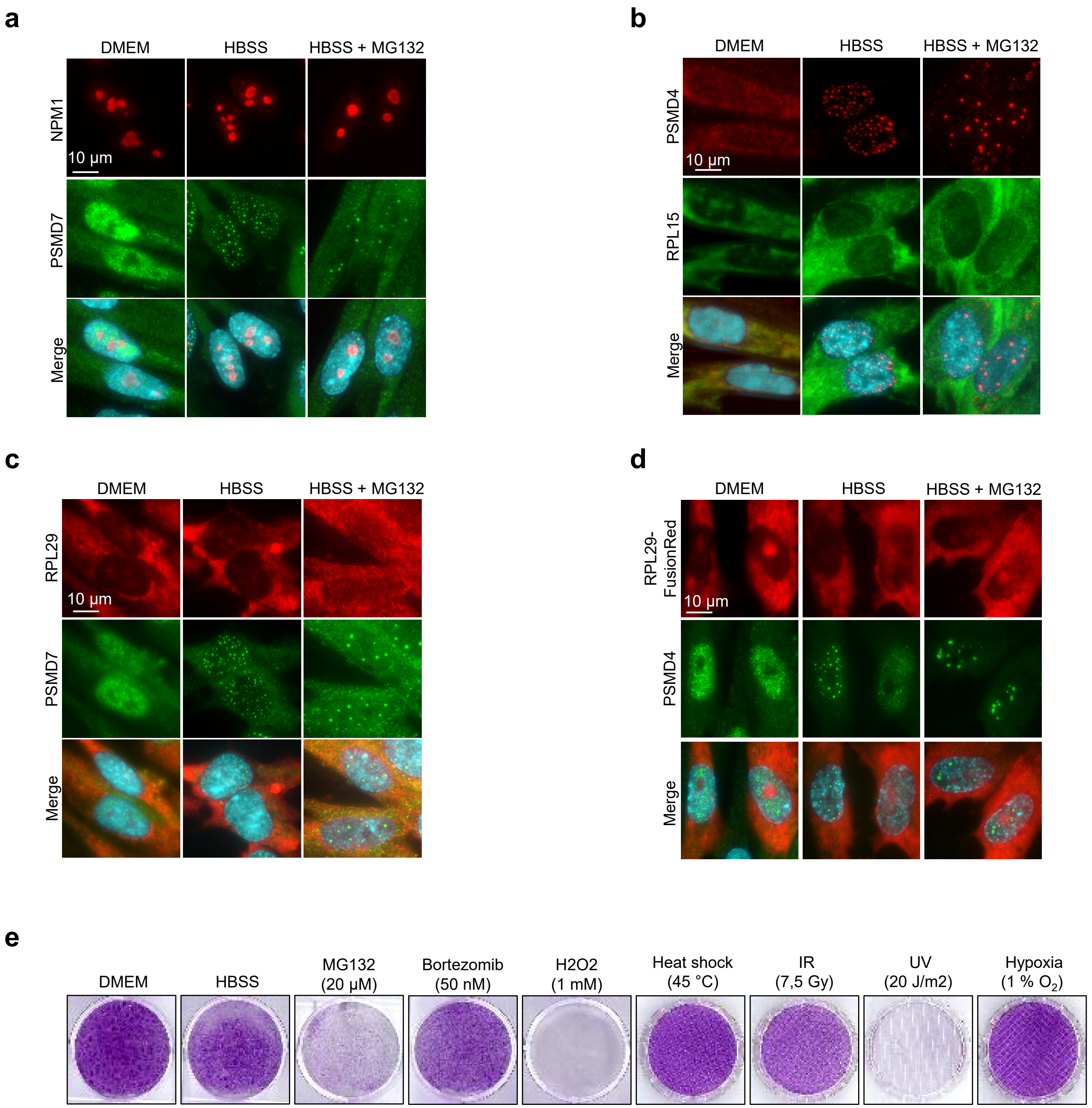


**Supplementary Figure 3**

**Supplementary Figure 3. SIPAN are not enriched with ribosomal proteins. a)** Nutrient deprivation does not induce changes in NPM1 localisation. IMR90 cells were incubated in HBSS or HBSS plus 10 μM MG132 for 6 hrs. **b, c)** RPL15 and RPL29 do not form foci in IMR90 cells after nutrient deprivation. Nutrient-deprived cells are treated with or without 10 μM MG132. **d)** Overexpression of RPL29-FusionRed in IMR90 cells followed by treatments with HBSS or HBSS plus 10 μM MG132, for 6 hrs, do not result in RPL29 foci formation. **e)** IMR90 cells were treated with various chemical agents or physical conditions and analyzed for SIPAN formation, as shown in Figure 3e. To ensure treatment efficacy, treated cells were also incubated in normal culture medium for 1 week and then stained with crystal violet. All panels are representative from 2 independent experiments.

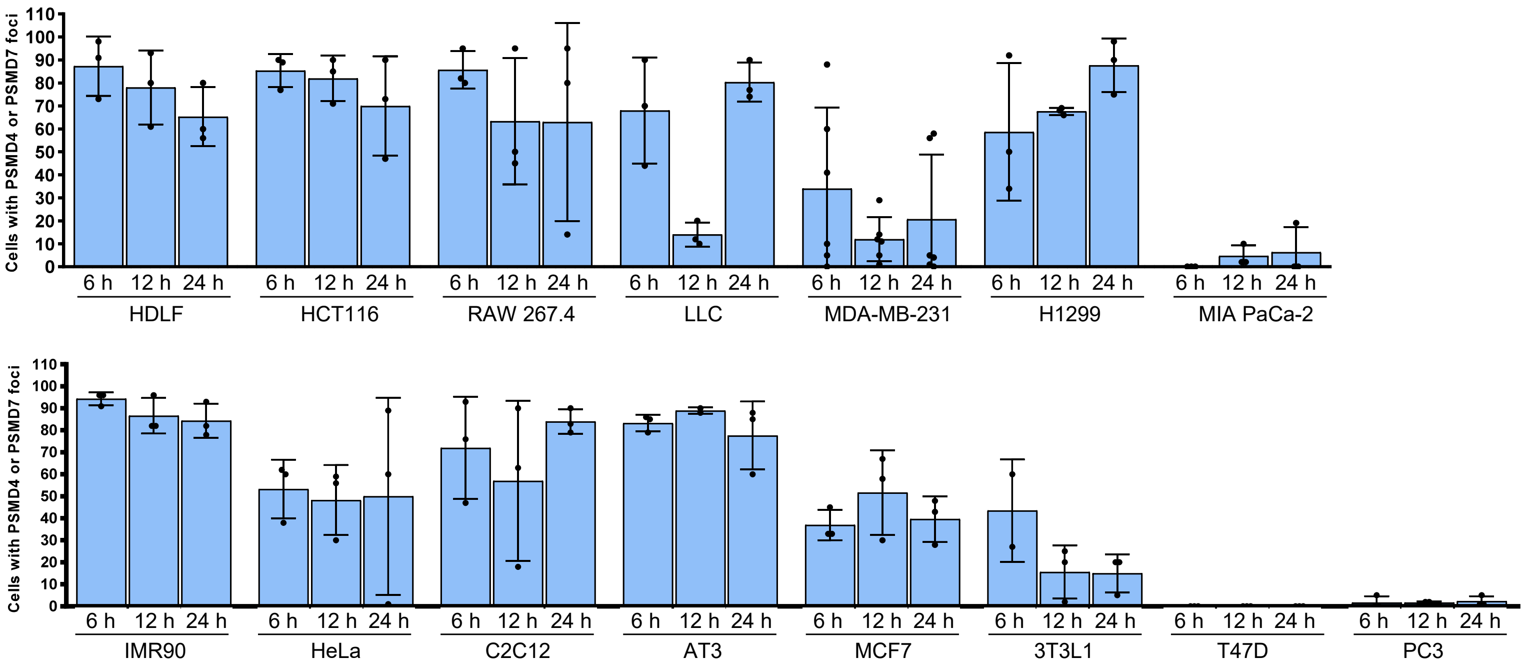


**Supplementary Figure 4**

**Supplementary Figure 4. SIPAN are observed in multiple cell lines and certain cancer cells have reduced ability to form SIPAN.** Multiple cell line were incubated in HBSS for the indicated time points, as shown in Figure 4a. Cells with more than 10 foci are counted. Data represent the mean +/- SD (n=3 independent experiments). Source data are provided as a Source Data file.

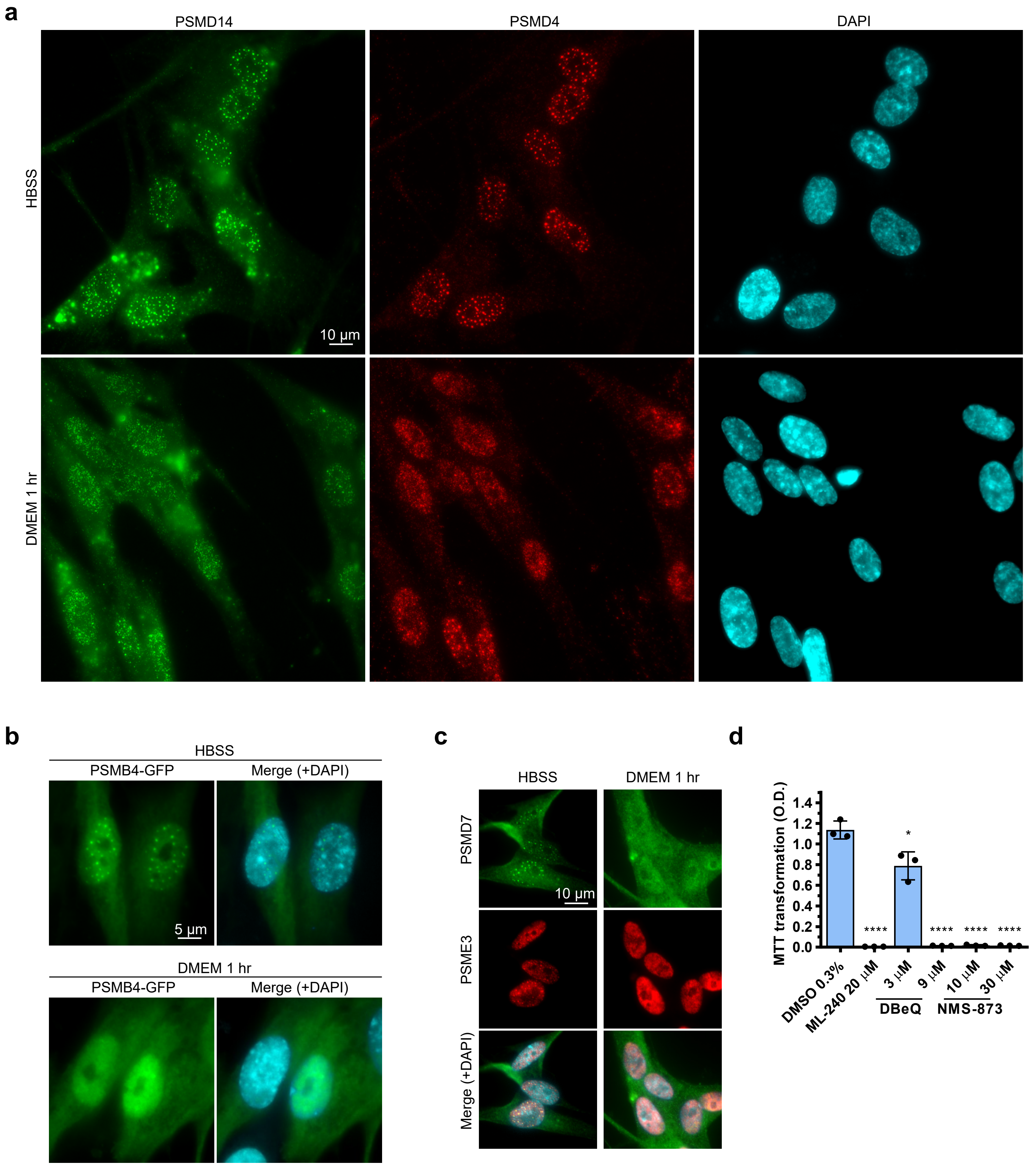


**Supplementary Figure 5**

**Supplementary Figure 5. Rapid dissipation of SIPAN after nutrient replenishment. a-c**) SIPAN are reversible. Foci formation is induced following incubation of IMR90 cells in HBSS for 6 hrs and the cells were then replenished with fresh culture medium (DMEM supplemented with 10% FBS and glutamine) for 1 hr and harvested for immunodetection of PSMD14 and PSMD4 (**a**), direct detection of PSMB4-GFP fluorescence (**b**), or immunodetection of PSMD7 and PSME3 (**c**). SIPAN dissipate as quickly as 1 hr after adding fresh culture medium (Representative from 5 independent experiments). **d**) Control for VCP inhibition (related to Figure 5o). Inhibition of VCP results in cell death. IMR90 cells were incubated in HBSS for 24 hrs in the presence of various inhibitors and harvested for MTT viability assay (n=3 independent experiments). Data in **d** represent the mean +/- SD. Cells with more than 10 foci are counted (**d**). *P<0.05; **P<0.01; ***P<0.001; ****P<0.0001; ns: not significant; 2-sided unpaired Student's t-test used in **d**. Source data are provided as a Source Data file.

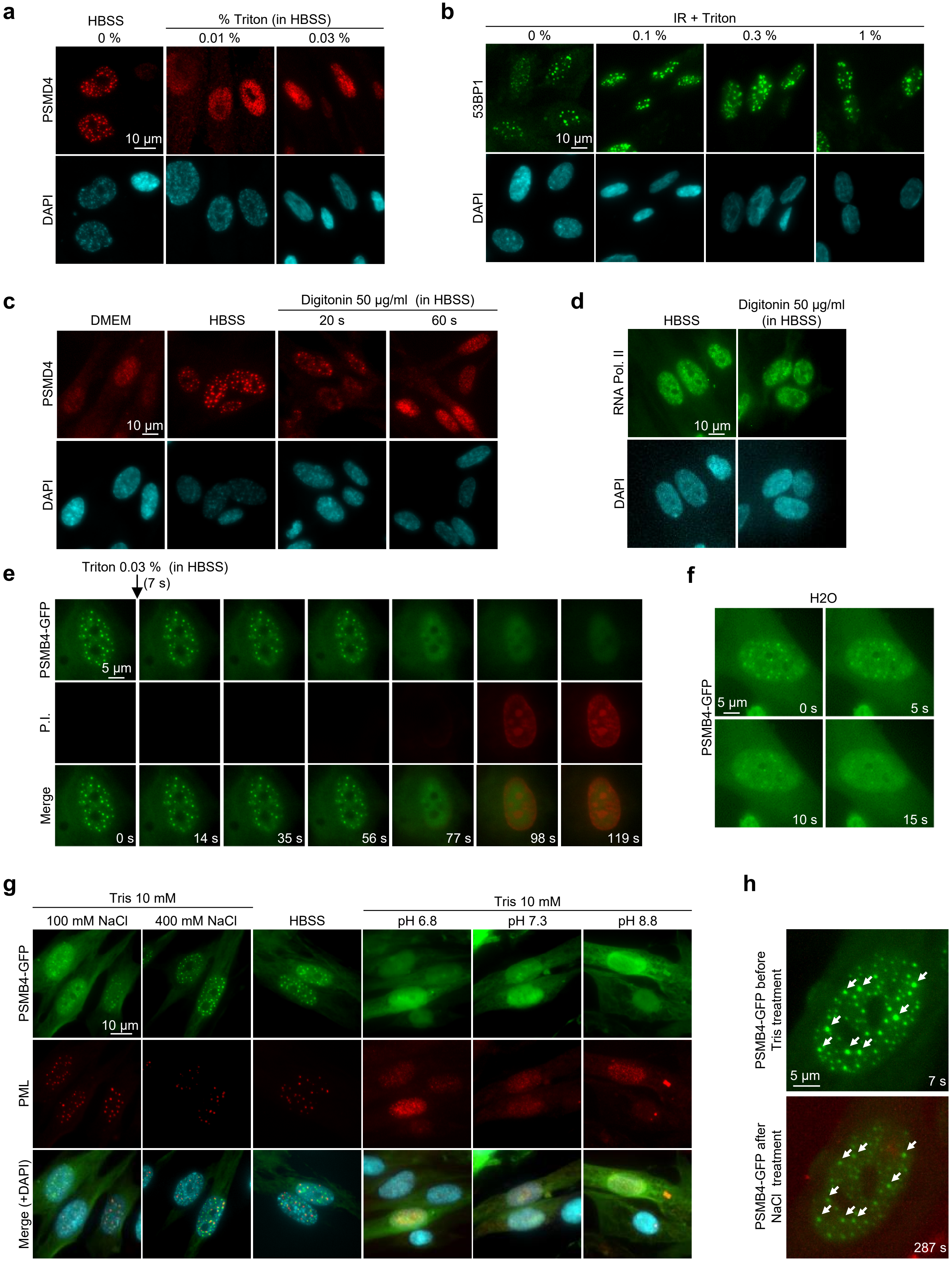


**Supplementary Figure 6**

**Supplementary Figure 6. SIPAN are highly responsive to physicochemical perturbations of the cell environment. a,b**) SIPAN dissipate rapidly following incubation of the cells with very low concentration of Triton X-100 detergent. 53BP1 DNA repair foci are not displaced with a concentration of Triton X-100 100 times more than that used to dissipate SIPAN. **a**) IMR90 cells with preformed proteasome foci were treated with Triton X-100 in HBSS for 2 min and then harvested for PSMD4 immunostaining. **b**) For 53BP1 foci, IMR90 cells were treated with ionizing radiations for 4 hrs and then used for detergent treatment in HBSS for 2 min before immunostaining. **c**) SIPAN become dissipated rapidly following incubation of the cells with Digitonin detergent. IMR90 cells were first treated for 8 hrs to allow SIPAN formation and then treated with Digitonin in HBSS for the indicated times. Cells were then harvested for immunostaining. **d**) RNA Polymerase II staining is not affected by Digitonin treatment. IMR90 cells with preformed SIPAN were treated with Digitonin in HBSS for 2 min and then harvested for RNA Polymerase II immunostaining. **e**) Live-cell imaging of PSMB4-GFP in IMR90 cells following addition of detergent. IMR90 cells were incubated in HBSS for 8 hrs to allow SIPAN formation, then treated with 0.03 % Triton X-100 in the presence of 50 µg/ml of propidium iodide (PI). **f**) Live imaging of PSMB4-GFP in IMR90 cells following addition of water. IMR90 cells were incubated in HBSS for 8 hrs to allow SIPAN formation, then treated as indicated. **g**) Concomitant detection of SIPAN (PSMB4-GFP) and PML bodies (PML staining) in IMR90 cells following addition of various buffers. Cells were incubated in HBSS for 8 hrs to allow SIPAN formation and then treated with various Tris buffers for 2 min before fixation and immunostaining. **h**) Merge of PSMB4-GFP before treatment of IMR90 with hypotonic buffer and after addition of 200 mM of NaCl (magnification from Figure 6d). All panels are representative from 3 independent experiments.

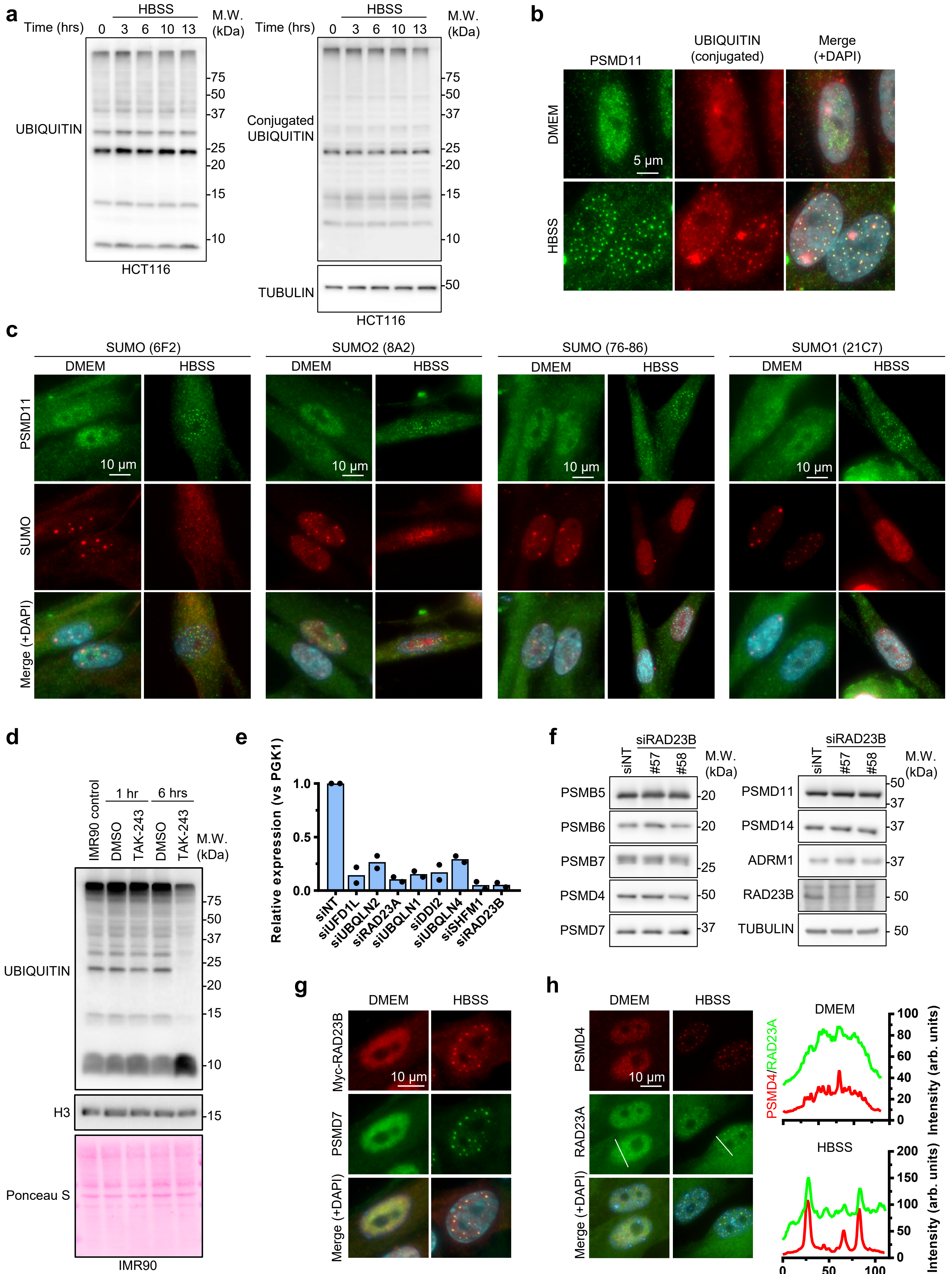


**Supplementary Figure 7**

**Supplementary Figure 7. SIPAN colocalize with conjugated ubiquitin, but not with SUMO. a)** Levels of ubiquitin and conjugated ubiquitin following nutrient deprivation in HCT116 cells. Cells were incubated in HBSS solution and harvested at different time points for immunoblotting with anti-ubiquitin or anti-conjugated ubiquitin (FK2) antibodies (Representative from 2 independent experiments). **b)** Immunostaining showing co-localization between proteasome foci and conjugated ubiquitin (FK2 antibody). IMR90 cells were treated for 6 hrs with HBSS and harvested for immunofluorescence (Representative from 3 independent experiments). **c**) SUMO does not co-localize with PSMD11 in SIPAN following nutrient starvation. IMR90 cells were incubated in HBSS for 6 hrs and harvested for immunostaining as indicated. Several antibodies against various SUMO epitopes were used (Representative from 3 independent experiments). **d)** To control for the efficacy of TAK-293-mediated UBA1 inhibition (figure 7g-j), IMR90 cells were treated with TAK-293 for 1 hr and 6 hrs and harvested to detect protein ubiquitination (Representative from 2 independent experiments). **e)** qRT-PCR analysis of proteasome shuttling factors after depletion by siRNA in IMR90 cells to verify efficacy of siRNAs used in figure 7I (n=1 experiment). **f**) Depletion of RAD23B does not affect the protein levels of components of the proteasome. Following siRNA transfection, IMR90 cells were harvested for immunoblotting (n=3 experiments). **g)** Overexpressed RAD23B is assembled in SIPAN following nutrient starvation. Following lentiviral infection, IMR90 cells were incubated in HBSS for 6 hrs and harvested for immunostaining for Myc-RAD23B (anti-Myc) and PSMD7 (Representative from 3 independent experiments). **h)** RAD23A forms foci with PSMD4 following nutrient deprivation. IMR90 were treated with HBSS for 6 hrs and harvested for immunostaining. Right panels, Diagrams show co-localization between RAD23A and PSMD4 (Representative from 3 independent experiments). Source data are provided as a Source Data file.

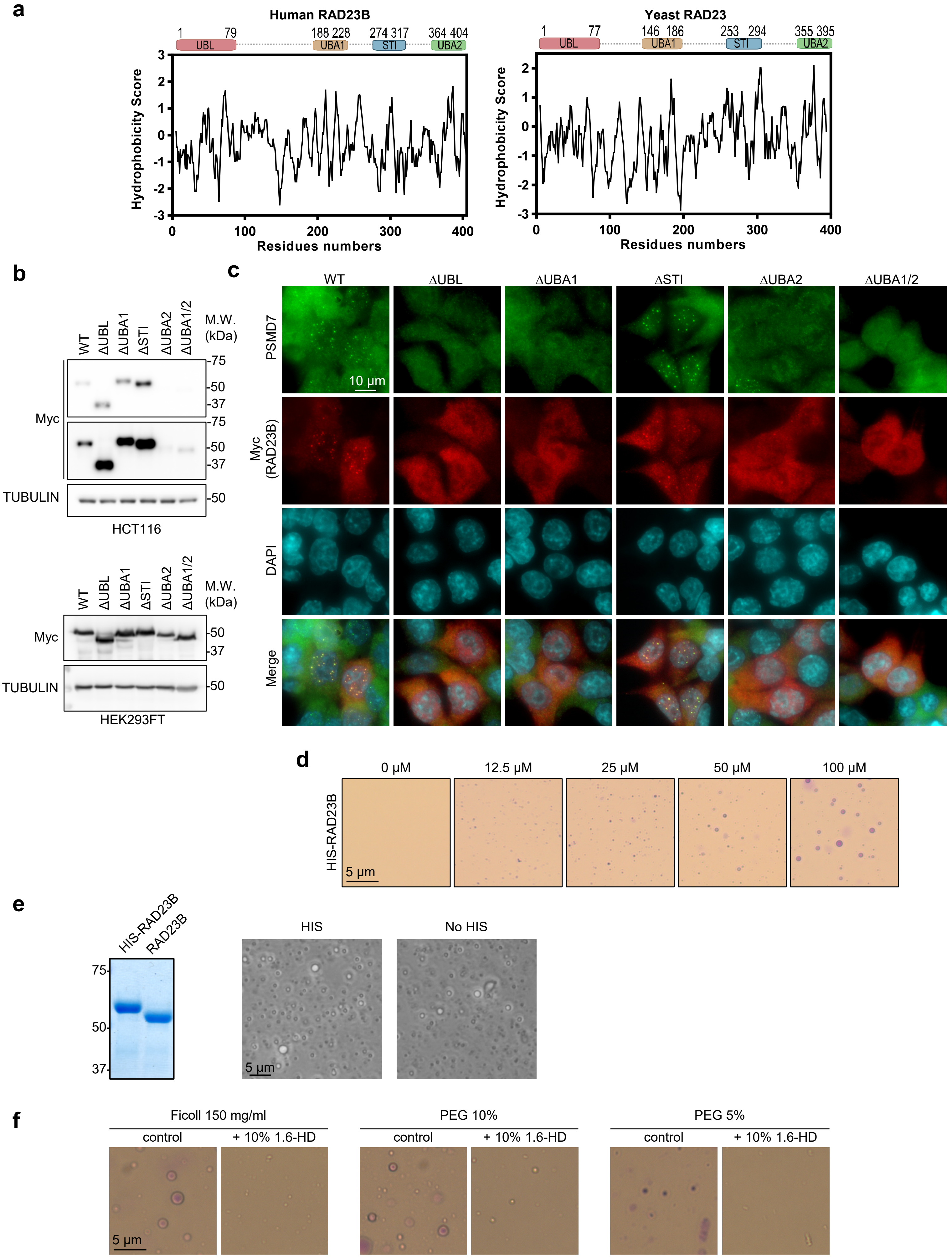


**Supplementary Figure 8**

**Supplementary Figure 8. Liquid-Liquid Phase separation of RAD23B in vitro. a)** Diagrams showing hydrophobicity profiles of human RAD23B and yeast RAD23 were derived using Kyte-Doolittle hydropathy algorithm. **b)** Expression levels of RAD23B mutants in HCT116 and HEK293FT (n=2 experiments). **c)** Representative images presented in figure 8c (Representative from 4 independent experiments). **d)** Formation of droplet is dependent on RAD23B concentration. Increasing concentration of His-RAD23B was mixed with Ficoll and droplets were stained and imaged immediately after. Crystal violet was used to stain the droplets (Representative from 3 independent experiments). **e)** His tag does not interfere with RAD23B droplets formation. His tag of His-RAD23B was cleaved with TEV protease enzyme and the purified protein was mixed with Ficoll to induce phase separation (Representative from 3 independent experiments). **f)** 1,6-Hexanediol inhibits RAD23B droplets formation induced with different crowding agents. His-RAD23B was mixed with PEG (5% or 10 %) or Ficoll (150 mg/ml) and droplets formation was stained by crystal violet analyzed by microscopy (Representative from 3 independent experiments). Source data are provided as a Source Data file.

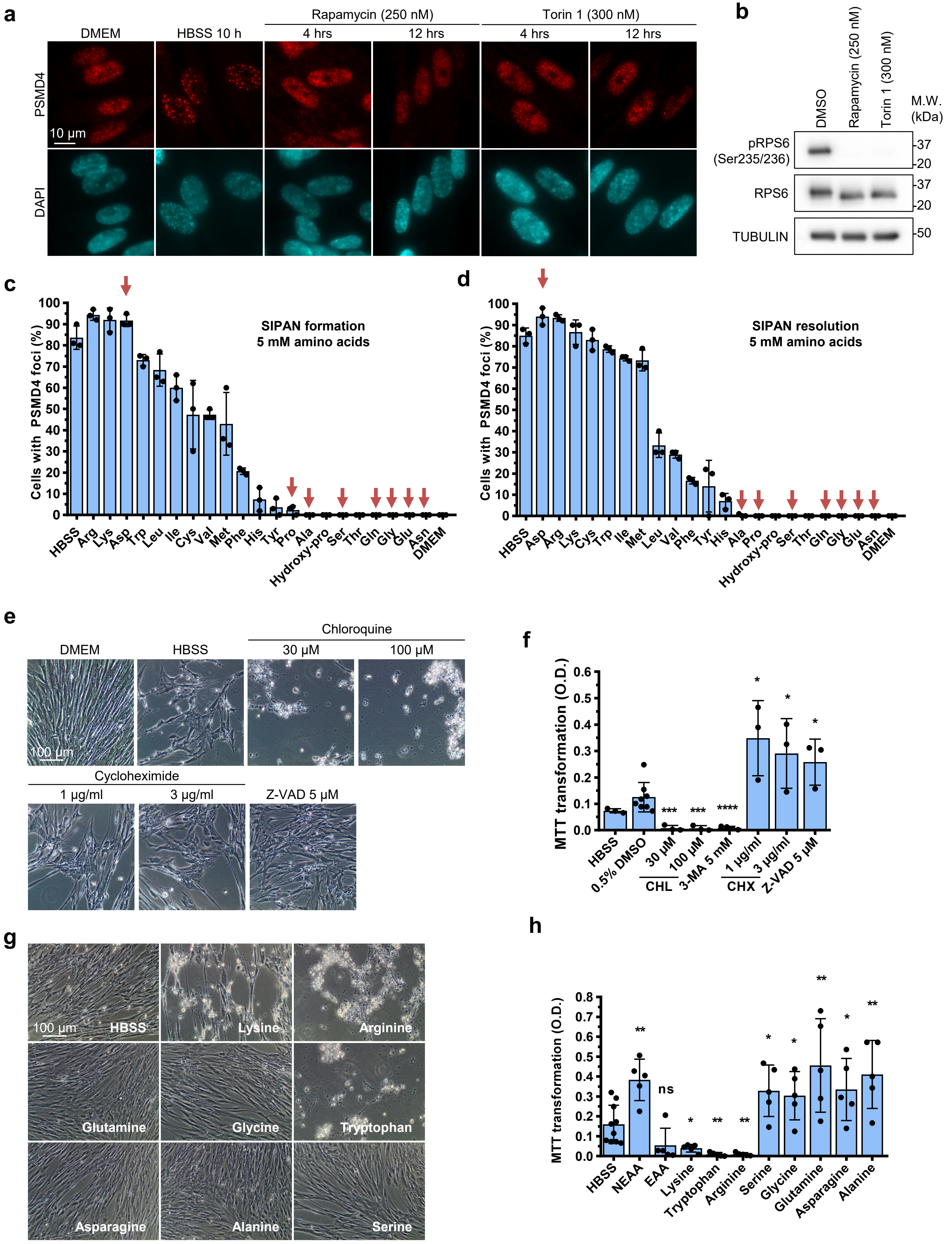


**Supplementary Figure 9**

**Supplementary Figure 9. Non-Essential Amino Acids (NEAA) inhibit SIPAN formation and protect from cell death that results from nutrient depletion. a)** Inhibition of mTOR does not result in SIPAN formation. IMR90 cells were incubated with the inhibitors as indicated and harvested for immunostaining (Representative from 3 independent experiments). **b)** Controls of mTOR inhibition. IMR90 cells were treated with mTOR inhibitors and harvested for immunoblotting with antibodies against the ribosomal protein S6 and its phosphorylated form, as indicated (Representative from 2 independent experiments). **c,d)** The majority of NEAA prevent SIPAN formation and promote their resolution. Left, IMR90 cells were incubated 5 mM concentration of individual amino acids in HBSS solution and harvested after 6 hrs for immunostaining. Right, SIPAN formation is induced and then cells were replenished with HBSS containing 5 mM concentration of individual amino acids. Cells were harvested after 1 hr for immunostaining. Red arrows represent NEAA (n=3 independent experiments). **e,f**) Chemical treatments that prevent SIPAN formation result in increased cell survival, whereas those that promote their formation result in increased cell death. IMR90 cells were incubated in HBSS for 48 hrs in the presence of various inhibitors and harvested for phase contrast imaging (**e**) and MTT viability assay (**f**) (n=3 independent experiments). **g,h**) Effects of Essential Amino Acids (EAA) and NEAA on cell viability. IMR90 cells were incubated in HBSS for 48 hrs in the presence of individual amino acids and harvested for phase contrast imaging (**g**) and MTT viability assay (**h**) (n=5 independent experiments). NEAA increase cell survival relative to EAA or HBSS alone. Data in **c**, **d**, **f** and **h** represent the mean +/- SD. *P<0.05; **P<0.01; ***P<0.001; ****P<0.0001; ns: not significant; 2-sided unpaired Student’s t-test used in **f** and **h**. Source data are provided as a Source Data file.

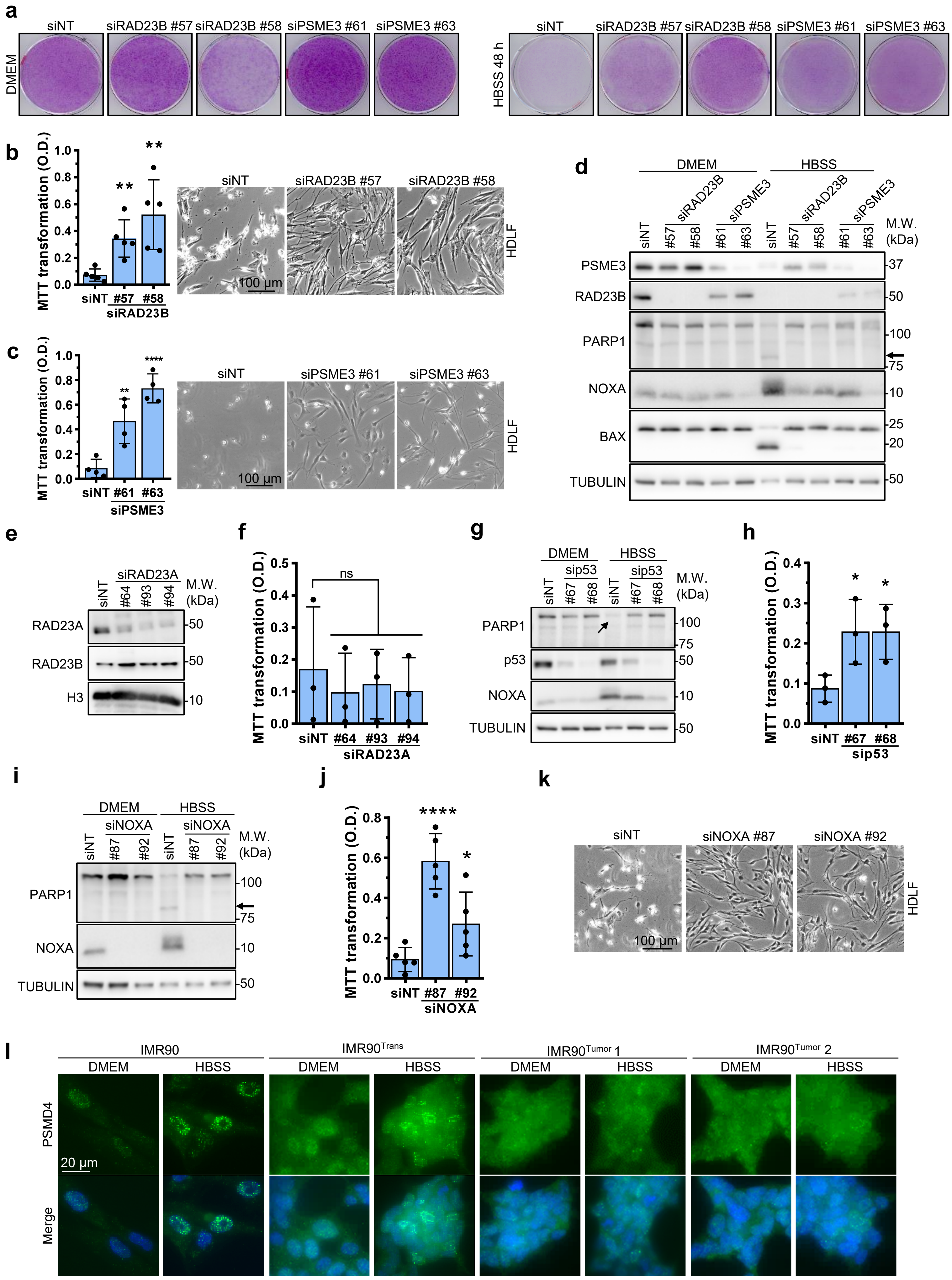


Supplementary Figure 10

**Supplementary Figure 10. Inhibition of RAD23B or PSME3 promotes cell survival in response to amino acid deprivation. a)** Following siRNA transfection, IMR90 cells were incubated in HBSS for 48 hrs and then incubated for one week in normal culture medium and harvested for crystal violet staining. Note that IMR90 cells in the DMEM condition were diluted 10 times before plating to avoid confluency (Representative from 3 independent experiments). **b-c)** Depletion of RAD23B (n=5 independent experiments) or PSME3 (n=4 independent experiments) results in increased cell survival following nutrient deprivation. Three days following siRNA transfection, HDFL cells were incubated in HBSS for 24 hrs and harvested for MTT viability assay and phase contrast imaging**. d**) Depletion of RAD23B or PSME3 prevents NOXA and BAX induction. After siRNA depletion of RAD23B or PSME3, HDLF cells are harvested for immunoblotting (Representative from 3 independent experiments). **e,f)** RAD23A depletion does not promote cell survival. Following RAD23A depletion by siRNA, IMR90 cells were harvested for Western blot (**e**) and MTT viability essay (**f**) (n=3 independent experiments). **g,h**) p53 depletion promotes IMR90 survival in response to amino acid depletion. IMR90 cells were incubated in HBSS for 6 hrs after depletion of p53 for (**g**) Western blotting and (**h**) MTT assay (n=3 independent experiments). **i-k)** Inhibition of the pro-apoptotic protein NOXA promotes HDFL cell survival after 24 hrs incubation in HBSS. Following NOXA depletion by siRNA, cells were treated and harvested for (**i**) Western blotting, (**j**) MTT assay and (**k**) phase contrast (n=5 independent experiments). **l**) Representative pictures of cells presented in figure **10l**. Data in **b**, **c**, **f, h** and **j** represent the mean +/- SD. *P<0.05; **P<0.01; ***P<0.001; ****P<0.0001; ns: not significant; 2-sided unpaired Student’s t-test used in **b**, **c**, **f**, **h** and **j**. Source data are provided as a Source Data file.

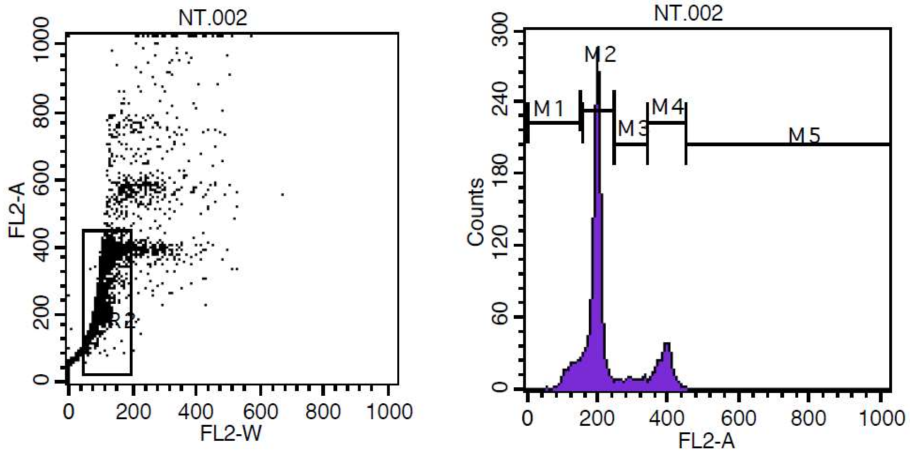


**Supplementary Figure 11**. Example of gating from Figure 10e showing gating strategy used in Figure 10e and Supplementary Figure 1b.

**Supplementary Table 1**

| Chemicals, Peptides, and Recombinant Proteins | Source | Catalog Number |
|---|---|---|
| MG132 | Sigma | C221 |
| ML-240 | Cayman | #17373 |
| NMS-873 | Cayman | #17674 |
| Rapamycin | Selleckchem | S1039 |
| Torin 1 | Selleckchem | S2827 |
| MEM Amino Acids Solution solution, 50X | Wisent | #321-010-EL |
| NEAA (MEM Non-essential Amino Acids Solution, 100X) | Wisent | #321-011-EL |
| Chloroquine diphosphate | Bioshop | CHL919.25 |
| Cycloheximide | Bioshop | CYC003.1 |
| Hydrogen peroxide solution | Sigma | #216763 |
| Digitonin | Sigma | DX1390-3 |
| Z-VAD-FMK | Selleckchem | S7023 |
| DBeQ | N/A | N/A |
| b-AP15 | Calbiochem | #662140 |
| 3-Methyladenine | Cayman | #13242 |
| Dextran 60000-90000 | ICN | #101513 |
| PEG 6000 | Alfa Aesar | #A17541 |
| 1,6-Hexanediol | Aldrich | #240117 |
| L-Alanine | Bioshop | ALA001.25 |
| L-Arginine hydrochloride | Bioshop | ARG006.25 |
| L-Asparagine anhydrous | Bioshop | ASP001.25 |
| L-Aspartic acid | Bioshop | ASP003.25 |
| L-Cysteine | Bioshop | CYS555.25 |
| L-Cystine | Bioshop | CYS400.25 |
| L-Glutamic acid | Bioshop | GLU202.100 |
| L-Glutamine | Bioshop | GLU102.25 |
| Glycine | Fisher | BP381-5 |
| L-Histidine monohydrochloride monohydrate | Bioshop | HIS200.25 |
| L-4-Hydroxyproline | Bioshop | HYP686.10 |
| L-Isoleucine | Bioshop | ISO910.25 |
| L-Leucine | Bioshop | LEU222.25 |
| L-Lysine monohydrochloride | Bioshop | LYS202.500 |
| L-Methionine | Bioshop | MET222.25 |
| L-Phenylalanine | Bioshop | PHA302.25 |
| L-Proline | Bioshop | PRO222.25 |
| L-Serine | Bioshop | SER333.25 |
| L-Threonine | Bioshop | THR002.25 |
| L-Tryptophan | Bioshop | TRP100.25 |
| L-Tyrosine | Bioshop | TYR333.25 |
| L-Valine | Bioshop | VAL201.25 |
| TAK-243 (MLN7243) | Selleckchem | S8341 |
| Me4BodipyFL-Ahx3Leu3VS (Proteasome Activity Probe) | BostonBiochem | I-190 |
| Collagenase, type 3 | Worthington | LS004180 |

| | | |
|---|---|---|
| DNase I | New England BioLabs | #M0303 |
| ECM Gel from Engelbreth-Holm-Swarm murine sarcoma (Matrigel) | Sigma-Aldrich | #E6909 |
| DMEM/HAM'S F-12 50/50 MIX | Wisent | #319-085-CL |

**Supplementary Table 2**

| Target | siRNA sequences | Names |
|---|---|---|
| DDI2 | GAAUCUACCUCACUGGGAA | SASI_Hs01_00215367 |
| DDI2 | GUUCCAUCGACCUGAAGAA | SASI_Hs02_00359727 |
| RAD23A | CAGGAGAACCCUCAGCUUU | SASI_Hs01_00145993 |
| RAD23A | CAGUUCAUCCAGAUGCUGA | SASI_Hs02_00338864 |
| RAD23B | GAAGCUAUAGAAAGGUUAA | SASI_Hs01_00198057 |
| RAD23B | CUAACUUGUUCACUGGAUU | SASI_Hs01_00198058 |
| RAD23B | GAAUCAGCCUCAGUUUCAA | SASI_Hs01_00198059 |
| RAD23B | CUUGGUAUAAGGUAGUAGA | SASI_Hs01_00198061 |
| SHFM1 | GGAUGACUUCUCUAAUCA | SASI_Hs01_00017254 |
| SHFM1 | CACAUGUCUGGGAGGAUAA | SASI_Hs01_00017255 |
| UBQLN1 | CUGAAAUGAUGGUCCAGAU | SASI_Hs01_00157571 |
| UBQLN1 | GACUUACUGUUCACCUUGU | SASI_Hs01_00157573 |
| UBQLN2 | CAUGUACACUGACAUUCAA | SASI_Hs01_00115238 |
| UBQLN2 | CCUAUUUCCACAAAUAGC | SASI_Hs01_00115239 |
| UBQLN4 | CAAACAGCAGGGUGACUUU | SASI_Hs01_00085653 |
| UBQLN4 | CUGAUCUGAUGCGUCACAU | SASI_Hs01_00085656 |
| UFD1L | GGAAGAAAGCCCUAAGUGA | SASI_Hs01_00153646 |
| UFD1L | GACUUUGUAGUUGUAUGCU | SASI_Hs01_00153648 |
| PSMB5 | GACAGUGAAGGGAACCGGA | SASI_Hs01_00076892 |
| PSMB5 | CAUGGUGUAUCAGUACAAA | SASI_Hs01_00076894 |
| PSMB6 | GUCUGCAAUUCACUGCCAA | SASI_Hs01_00017705 |
| PSMB6 | CUACAUCUAUGGCUAUGUU | SASI_Hs01_00017706 |
| PSMB7 | CACUUCAUAUCUCCUAAUA | SASI_Hs02_00334458 |
| PSMB7 | CCAAGAAUCUGGUGAGCGA | SASI_Hs01_00241662 |
| PSMD14 | GUGAUUGAUGUGUUUGCUA | SASI_Hs01_00024446 |
| PSMD14 | CAGAAGAUGUUGCUAAAUU | SASI_Hs01_00024447 |
| PSMD11 | CCAAGUUGUAUGAUAACUU | SASI_Hs01_00123192 |
| PSMD11 | GCAUUUGAGGGUUAUGACU | SASI_Hs01_00123189 |
| PSMD4 | CCAAUUCUACCCUGCUCCU | SASI_Hs01_00179690 |
| PSMD4 | GCACUAUGGUGUGUGUGGA | SASI_Hs01_00179692 |
| PSMD7 | CACUUGUUACGAGAUAUCA | SASI_Hs02_00334492 |
| PSMD7 | CGUGUUGUUGGUGUGCUUU | SASI_Hs02_00334491 |
| PSME3 | GAAGGAAAGUGCUAGGUGU | SASI_Hs01_00137661 |
| PSME3 | CUAAUGAAACUCUCAUCUA | SASI_Hs01_00137663 |
| PMAIP1 (NOXA) | CGUUUCAUCAAUUUGAAGA | SASI_Hs01_00136187 |
| PMAIP1 (NOXA) | CAUUCUUUGGGAUGAGAGA | SASI_Hs01_00136192 |
| TP53 | GUCUUUGAACCCUUGCUUG | SASI_Hs02_00302767 |
| TP53 | CCACUACAACUACAUGUGU | SASI_Hs02_00302768 |

**Supplementary Table 3**

| Antibodies | Source | Catalog number | Western blot | Immuno-fluorescence | Antigen retrieval |
|---|---|---|---|---|---|
| Mouse monoclonal anti-Fibrillarin | Santa Cruz | SC-166001 | N.A. | 1/1000 | no |
| Mouse monoclonal anti-ADRM1 | Santa Cruz | SC-166754 | 1/1000 | 1/1000 | no |
| Mouse monoclonal anti-FK2 | Millipore | #04-263 | 1/1000 | N.A. | no |
| Mouse monoclonal anti-LDH | Santa Cruz | SC-133123 | 1/1000 | 1/1000 | no |
| Mouse monoclonal anti-PA28γ (PSME3) | Santa Cruz | SC-136025 | N.A. | 1/1000 | no |
| Mouse monoclonal anti-SC35 | Santa Cruz | SC-53518 | N.A. | 1/1000 | no |
| Mouse monoclonal anti-PSMB1 | Santa Cruz | SC-374405 | N.A. | 1/1000 | no |
| Mouse monoclonal anti-PSMB2 | Santa Cruz | SC-365725 | N.A. | 1/1000 | no |
| Mouse monoclonal anti-PSMB4 | Santa Cruz | SC-390878 | N.A. | 1/1000 | no |
| Rabbit polyclonal anti-PSMB5 | Bethyl | #A303-847A | 1/1000 | 1/500 | yes |
| Rabbit monoclonal anti-PSMB6 | Cell Signaling | #13267 | 1/1000 | 1/500 | yes |
| Rabbit monoclonal anti-PSMB7 | Cell Signaling | #13207 | 1/1000 | 1/500 | yes |
| Rabbit polyclonal anti-PSMD11 | Bethyl | #A302-751A | 1/1000 | 1/1000 | no |
| Rabbit monoclonal anti-PSMD14 | Cell Signaling | #4197S | 1/1000 | 1/2000 | no |
| Mouse monoclonal anti-PSMD4 | Santa Cruz | SC-393546 | 1/1000 | 1/500 | no |
| Rabbit polyclonal anti-UCH37 | Bethyl | A304099A | N.A. | 1/1000 | no |
| Rabbit polyclonal anti-PSMD7 | Bethyl | A303-828A | 1/1000 | 1/1000 | yes |
| Mouse monoclonal anti-RAD23B | Santa Cruz | SC-166507 | 1/1000 | 1/1000 | yes |
| Mouse monoclonal anti-Tubulin | Santa Cruz | SC-23948 | 1/1000 | N.A. | no |
| Mouse monoclonal anti-Ub | Santa Cruz | SC-8017 | 1/1000 | 1/1000 | no |
| Rabbit polyclonal anti-c-fos | Santa Cruz | SC-7202 | 1/1000 | N.A. | no |
| Mouse monoclonal anti-PARP1 | Santa Cruz | SC-8007 | 1/1000 | N.A. | no |
| Mouse monoclonal anti-p53 | Santa Cruz | SC-126 | 1/1000 | N.A. | no |
| Mouse monoclonal anti-c-jun | Santa Cruz | SC-74543 | 1/1000 | N.A. | no |
| Rabbit polyclonal anti-53BP1 | Santa Cruz | SC-22760 | 1/1000 | 1/1000 | no |
| Mouse monoclonal anti-PML | Santa Cruz | SC-966 | N.A. | 1/1000 | no |
| Mouse monoclonal anti-H3 | Cell Signaling | #14269S | 1/20000 | N.A. | no |
| Mouse monoclonal anti-RNA polymerase II | Millipore | #05-952 | 1/1000 | N.A. | no |
| Mouse monoclonal anti-SUMO1 21C7 | DSHB | SUMO1 21C7 | 1/250 | N.A. | no |
| Mouse monoclonal anti-SUMO1 76-86 | DSHB | SUMO1 76-86 | 1/250 | N.A. | no |
| Mouse monoclonal anti-SMUO-2 8A2 | DSHB | SMUO-2 8A2 | 1/250 | N.A. | no |
| Mouse monoclonal anti-SUMO 6F2 | DSHB | SUMO 6F2 | 1/250 | N.A. | no |
| Mouse monoclonal anti-caspase 3 | Santa Cruz | SC-56053 | 1/1000 | N.A. | no |
| Mouse monoclonal anti-USP14 | Santa Cruz | SC-100630 | 1/1000 | N.A. | no |
| Rabbit monoclonal anti-S6 ribosomal protein | Cell Signaling | #2217S | 1/1000 | N.A. | no |
| Rabbit monoclonal anti-P-S6 ribosomal protein | Cell Signaling | #4858S | 1/1000 | N.A. | no |
| Rabbit polyclonal anti-P-4EBP1 | Cell Signaling | #9459S | 1/1000 | N.A. | no |
| Rabbit monoclonal anti-4EBP1 | Cell Signaling | #9644S | 1/1000 | N.A. | no |
| Mouse monoclonal anti-MYC | This paper | homemade | 1/1000 | N.A. | no |
| Mouse monoclonal anti-MCL-1 | Santa Cruz | SC-74436 | 1/500 | N.A. | no |
| Mouse monoclonal anti-NOXA | Santa Cruz | SC-56169 | 1/500 | N.A. | no |
| Mouse monoclonal anti-PUMA | Santa Cruz | SC-374223 | 1/500 | N.A. | no |
| Rabbit monoclonal anti-BAK | Cell Signaling | #5023T | 1/1000 | N.A. | no |
| Rabbit monoclonal anti-BAX | Cell Signaling | #12105T | 1/1000 | N.A. | no |
| Rabbit monoclonal anti-BIM | Cell Signaling | #2933T | 1/1000 | N.A. | no |

| | | | | | |
|---|---|---|---|---|---|
| Rabbit monoclonal anti-RAD23B | Cell Signaling | #13525 | 1/1000 | 1/1000 | yes |
| Mouse polyclonal anti-RPL29 | Abnova | H00006159-B01P | N.A. | 1/1000 | yes |
| Rabbit polyclonal anti-RPL15 | Proteintech | 16740-1-AP | N.A. | 1/1000 | yes |
| Mouse monoclonal anti-Phospho-H2AX (ser139) | Millipore | 05-636-I | 1/1000 | N.A. | no |
| Mouse monoclonal anti-HSP70 | Santa Cruz | SC-66048 | 1/1000 | N.A. | no |
| Mouse monoclonal anti-HIF1alpha | Santa Cruz | SC-13515 | 1/1000 | N.A. | no |
| Rabbit monoclonal Anti-Ubiquitin K48 | Abcam | ab140601 | 1/1000 | 1/1000 | no |
| Mouse monoclonal anti-NPM1 | Thermofisher | #32-5200 | N.A. | 1/1000 | no |
| Goat polyclonal anti-Rabbit Alexa fluor 594 | Invitrogen | A11012 | 1/1000 | N.A. | N.A. |
| Goat polyclonal anti-Mouse Alexa fluor 594 | Invitrogen | A11005 | 1/1000 | N.A. | N.A. |
| Goat polyclonal anti-Rabbit Alexa fluor 488 | Invitrogen | A11008 | 1/1000 | N.A. | N.A. |
| Goat polyclonal anti-Mouse Alexa fluor 488 | Invitrogen | A11029 | 1/1000 | N.A. | N.A. |
| Goat polyclonal anti-Mouse HRP | Jackson Immunoresearch | 115-036-003 | N.A. | 1/1000 | N.A. |
| Goat polyclonal anti-Rabbit HRP | Jackson Immunoresearch | 111-036-003 | N.A. | 1/1000 | N.A. |

**Supplementary Table 4**

| Name | Sequence (5'-3') |
|---|---|
| DDI2_forward | CAGGGATTGCCAAAGGAGTG |
| DDI2_reverse | TGCCGTTTAAGCATGTCCAG |
| RAD23A_forward | TCCACGCTAGTGACGGGCTCT |
| RAD23A_reverse | CAGGAATTCCCGTGAGCAGA |
| RAD23B_forward | GCTACTAGCCCAACAGCAAC |
| RAD23B_reverse | TGCTCTCGTTCATAGCCCAT |
| UFD1L_forward | CCAGGGTCTTCCAAAACCG |
| UFD1L_reverse | TGTTAAGTCGGCTGAGTTGGT |
| UBQLN1_forward | GATCATTCAGCTCAGCAAACA |
| UBQLN1_reverse | GTATTCAAACCCAAGCTACTCAGA |
| UBQLN2_forward | TGCCGCGGGAACTAACACTAC |
| UBQLN2_reverse | GGAGCTCAGAGAAGTTGGTCGA |
| UBQLN4_forward | CTGATCTGATGCGTCACATGAT |
| UBQLN4_reverse | ATTCCGAGCAAGCTCCATTGT |
| SHFM1_forward | CTGGGCTGGCTTAGATGAAGATG |
| SHFM1_reverse | CTTCTGGATGCTATGAAGTCTCC |
| PGK1_forward | GGTGGTGAAAGCCACTTCTA |
| PGK1_reverse | CTTTATCCTCCGTGTTCCATTT |
