# Supplementary material for: Starvation-induced proteasome assemblies in the nucleus link amino acid supply to apoptosis
Source: Nat Commun. 2021 Nov 30;12:6984. doi: 10.1038/s41467-021-27306-4 (PMC8633328; doi:10.1038/s41467-021-27306-4)
Supplement: Supplementary file 3 — Description of Additional Supplementary Files [file 41467_2021_27306_MOESM3_ESM.docx]

Description of Additional Supplementary Files

File name: Supplementary Movie 1

Description: Nutrient starvation induces the formation of proteasome foci in the nucleus of IMR90 cells. IMR90 cells stably expressing PSMB4-GFP were first incubated in HBSS for 1 h 30 and then used for live- cell fluorescence microscopy.

File name: Supplementary Movie 2

Description: Cell morphology during nutrient starvation of IMR90 cells. Differential interference contrast (DIC) live-cell imaging corresponding to Video 1.

File name: Supplementary Movie 3

Description: SIPAN fusion in vivo. Video extracted from Video 1 focusing on SIPAN fusion.

File name: Supplementary Movie 4

Description: SIPAN dissipate following treatment with detergent before propidium iodide entry. IMR90 cells stably expressing PSMB4-GFP were incubated in HBSS for 8 h to induce SIPAN formation and then cells were treated with HBSS containing 0.03 % of Triton X-100 detergent and 50 µg/ml of propidium iodide.

File name: Supplementary Movie 5

Description: SIPAN dissipate following treatment with 1,6-hexanediol. IMR90 cells expressing PSMB4-GFP were incubated in HBSS for 8 hours and used for live-imaging in the presence of 10% 1,6-Hexanediol.

File name: Supplementary Movie 6

Description: SIPAN dissipate following treatment with hypotonic buffer and recover immediately following salt replenishment. IMR90 cells stably expressing PSMB4-GFP were incubated in HBSS and then in Tris 10 mM pH 7.3 (SIPAN dissipation), followed by NaCl 200 mM in Tris 10 mM pH 7.3 (SIPAN recovery), respectively.

File name: Supplementary Movie 7

Description: Fluorescence recovery after photobleaching (FRAP) of SIPAN. HCT116 cells expressing PSMB4-GFP were incubated in HBSS for 6 h to induce SIPAN formation. Bleaching of PSMB4-GFP foci indicates that SIPAN appear rapidly in their original foci following bleaching.

File name: Supplementary Movie 8

Description: Fluorescence recovery after photobleaching (FRAP) of 53BP1 foci. HCT116 cells expressing GFP-53BP1 were treated with ionizing radiation for 4 h to induce double strand DNA break and 53BP1 foci formation. Bleaching of GFP-53BP1 indicates that very low recovery of fluorescence was observed for these foci.

File name: Supplementary Movie 9

Description: Fluorescence recovery after photobleaching (FRAP) of histone H2A domains. HCT116 cells expressing histone GFP-H2A were used to bleach histone H2A-rich chromatin domains. No apparent recovery of histone H2A fluorescence was observed.

File name: Supplementary Movie 10

Description: RAD23B droplets fusion events during phase separation in vitro. Purified RAD23B was mixed with Ficoll 400 and the mixture was deposited on a microscope slide. Fusions events were observed by live-cell light microscopy.

File name: Supplementary Movie 11

Description: Exhaustion of non-essential amino acid is responsible for SIPAN formation. IMR90 cells expressing PSMB4-GFP were incubated in HBSS in the presence of 100 µM of chloroquine and used for live-imaging. Inhibition of autophagy by chloroquine accelerates SIPAN formation

File name: Supplementary Movie 12

Description: Non-essential amino acids induce SIPAN resolution. IMR90 cells expressing PSMB4-GFP were incubated in HBSS for 8 hours and then treated with a mixture of NEAA and used for live-imaging.
