# Supplementary material for: Starvation-induced proteasome assemblies in the nucleus link amino acid supply to apoptosis
Source: Nat Commun. 2021 Nov 30;12:6984. doi: 10.1038/s41467-021-27306-4 (PMC8633328; doi:10.1038/s41467-021-27306-4)
Supplement: Supplementary file 16 — Reporting Summary [file 41467_2021_27306_MOESM16_ESM.pdf]

nature research

Corresponding author(s): El Bachir Affar

Last updated by author(s): Oct 15, 2021

Reporting Summary

Nature Research wishes to improve the reproducibility of the work that we publish. This form provides structure for consistency and transparency in reporting. For further information on Nature Research policies, see Authors & Referees and the Editorial Policy Checklist.

Statistics

For all statistical analyses, confirm that the following items are present in the figure legend, table legend, main text, or Methods section.

n/a	Confirmed	
☐	☒	The exact sample size (n) for each experimental group/condition, given as a discrete number and unit of measurement
☐	☒	A statement on whether measurements were taken from distinct samples or whether the same sample was measured repeatedly
☐	☒	The statistical test(s) used AND whether they are one- or two-sided<br>*Only common tests should be described solely by name; describe more complex techniques in the Methods section.*
☐	☒	A description of all covariates tested
☒	☐	A description of any assumptions or corrections, such as tests of normality and adjustment for multiple comparisons
☐	☒	A full description of the statistical parameters including central tendency (e.g. means) or other basic estimates (e.g. regression coefficient) AND variation (e.g. standard deviation) or associated estimates of uncertainty (e.g. confidence intervals)
☐	☒	For null hypothesis testing, the test statistic (e.g. F, t, r) with confidence intervals, effect sizes, degrees of freedom and P value noted<br>*Give P values as exact values whenever suitable.*
☒	☐	For Bayesian analysis, information on the choice of priors and Markov chain Monte Carlo settings
☒	☐	For hierarchical and complex designs, identification of the appropriate level for tests and full reporting of outcomes
☒	☐	Estimates of effect sizes (e.g. Cohen's d, Pearson's r), indicating how they were calculated

Our web collection on statistics for biologists contains articles on many of the points above.

Software and code

Policy information about availability of computer code

Data collection	ImageJ/FIJI (v1.53, https://imagej.net/Fiji)<br>Zen 2 (Zeiss)<br>GE SoftWoRx software (v7, GE Healthcare)<br>Fluoview (FV1000, Olympus)<br>CellSens software (v1.7.1, Olympus)<br>MultiGauge software<br>Azurec600 Imaging System (software version 1.9.7, Azure Biosystem)<br>CellQuestPro software (BD Biosciences)<br>KaleidaGraph (v4.5, Synergy)<br>MetaMorph (Molecular Devices)
Data analysis	Prism 8 (Graphpad)<br>ImageJ/FIJI (v1.53, https://imagej.net/Fiji)<br>FlowJo (v10.7)<br>Zen 2 (Zeiss)

For manuscripts utilizing custom algorithms or software that are central to the research but not yet described in published literature, software must be made available to editors/reviewers. We strongly encourage code deposition in a community repository (e.g. GitHub). See the Nature Research guidelines for submitting code & software for further information.

Data

Policy information about availability of data

All manuscripts must include a data availability statement. This statement should provide the following information, where applicable:

- Accession codes, unique identifiers, or web links for publicly available datasets
- A list of figures that have associated raw data
- A description of any restrictions on data availability

There are no applicable accession codes, unique identifiers or datasets that could be publicly available. Additional data that support the findings of this study are available from the corresponding author (El Bachir Affar) upon reasonable request.

Field-specific reporting

Please select the one below that is the best fit for your research. If you are not sure, read the appropriate sections before making your selection.

☒ Life sciences ☐ Behavioural & social sciences ☐ Ecological, evolutionary & environmental sciences

For a reference copy of the document with all sections, see nature.com/documents/nr-reporting-summary-flat.pdf

Life sciences study design

All studies must disclose on these points even when the disclosure is negative.

Sample size	No statistical method was used to predetermine sample size.<br>Cells are cultured and prepared to perform independent expriments for powerful statistical analysis and based on previous related studies in the research area.<br>1. For counts of cell with foci, we counted a minimum of 200 cells in independent experiments.<br>2. For measurements using the MTT test, we carried out at least 3 technical replicates in independent experiments.
Data exclusions	No samples were excluded from the analysis.
Replication	The number of experiment repeats is given in figure legends.
Randomization	Experiments were performed with cultured cell lines randomly seeded and randomly assigned for treatments. For RT-qPCR, western blot, and in vitro assay, cell conditions were known when we prepare the samples or set up the assays.
Blinding	The investigators were not blinded during data collection because the experiments did not require blinding and were readily controlled without blinding. All samples including controls were analysed in the same manner. For western blot, microscopy and in vitro assay, blinding was occasionally used.

Behavioural & social sciences study design

All studies must disclose on these points even when the disclosure is negative.

Study description	Not applicable
Research sample	Not applicable
Sampling strategy	Not applicable
Data collection	Not applicable
Timing	Not applicable
Data exclusions	Not applicable
Non-participation	Not applicable
Randomization	Not applicable

Ecological, evolutionary & environmental sciences study design

All studies must disclose on these points even when the disclosure is negative.

Study description	Not applicable
Research sample	Not applicable
Sampling strategy	Not applicable
Data collection	Not applicable
Timing and spatial scale	Not applicable
Data exclusions	Not applicable
Reproducibility	Not applicable
Randomization	Not applicable
Blinding	Not applicable

Did the study involve field work? ☐ Yes ☒ No

Reporting for specific materials, systems and methods

We require information from authors about some types of materials, experimental systems and methods used in many studies. Here, indicate whether each material, system or method listed is relevant to your study. If you are not sure if a list item applies to your research, read the appropriate section before selecting a response.

Materials & experimental systems

n/a	Involved in the study	
☐	☒	Antibodies
☐	☒	Eukaryotic cell lines
☒	☐	Palaeontology
☐	☒	Animals and other organisms
☒	☐	Human research participants
☒	☐	Clinical data

Methods

n/a	Involved in the study	
☒	☐	ChIP-seq
☐	☒	Flow cytometry
☒	☐	MRI-based neuroimaging

Antibodies

Antibodies used

Mouse monoclonal anti-Fibrillarin - Santa Cruz - B1 - SC-166001 - WB: N.A. - IF: 1/1000
Mouse monoclonal anti-ADRM1 - Santa Cruz - F12 - SC-166754 - WB: 1/1000 - IF: 1/1000
Mouse monoclonal anti-FK2 - Millipore - FK2 - #04-263 - WB: 1/1000 - IF: N.A.
Mouse monoclonal anti-LDH - Santa Cruz - H10 - SC-133123 - WB: 1/1000 - IF: 1/1000
Mouse monoclonal anti-PA28γ (PSME3) - Santa Cruz - 47 - SC-136025 - WB: N.A. - IF: 1/1000
Mouse monoclonal anti-SC35 - Santa Cruz - SC-35 - SC-53518 - WB: N.A. - IF: 1/1000
Mouse monoclonal anti-PSMB1 - Santa Cruz - D-9 - SC-374405 - WB: N.A. - IF: 1/1000
Mouse monoclonal anti-PSMB2 - Santa Cruz - H-3 - SC-365725 - WB: N.A. - IF: 1/1000
Mouse monoclonal anti-PSMB4 - Santa Cruz - H-3 - SC-390878 - WB: N.A. - IF: 1/1000
Rabbit polyclonal anti-PSMB5 - Bethyl - - #A303-847A - WB: 1/1000 - IF: 1/500
Rabbit monoclonal anti-PSMB6 - Cell Signaling - E1K9O - #13267 - WB: 1/1000 - IF: 1/500
Rabbit monoclonal anti-PSMB7 - Cell Signaling - E1L5H - #13207 - WB: 1/1000 - IF: 1/500
Rabbit polyclonal anti-PSMD11 - Bethyl - - #A302-751A - WB: 1/1000 - IF: 1/1000
Rabbit monoclonal anti-PSMD14 - Cell Signaling - D18C7 - #4197S - WB: 1/1000 - IF: 1/2000
Mouse monoclonal anti-PSMD4 - Santa Cruz - E-2 - SC-393546 - WB: 1/1000 - IF: 1/500
Rabbit polyclonal anti-UCH37 - Bethyl - - A304099A - WB: N.A. - IF: 1/1000
Rabbit polyclonal anti-PSMD7 - Bethyl - - A303-828A - WB: 1/1000 - IF: 1/1000
Mouse monoclonal anti-RAD23B - Santa Cruz - C-4 - SC-166507 - WB: 1/1000 - IF: 1/1000
Mouse monoclonal anti-Tubulin - Santa Cruz - B512 - SC-23948 - WB: 1/1000 - IF: N.A.
Mouse monoclonal anti-Ub - Santa Cruz - P4D1 - SC-8017 - WB: 1/1000 - IF: 1/1000
Rabbit polyclonal anti-c-fos - Santa Cruz - H-125 - SC-7202 - WB: 1/1000 - IF: N.A.
Mouse monoclonal anti-PARP1 - Santa Cruz - F-2 - SC-8007 - WB: 1/1000 - IF: N.A.
Mouse monoclonal anti-p53 - Santa Cruz - DO-1 - SC-126 - WB: 1/1000 - IF: N.A.
Mouse monoclonal anti-c-jun - Santa Cruz - G-4 - SC-74543 - WB: 1/1000 - IF: N.A.

Rabbit polyclonal anti-53BP1 - Santa Cruz - H-300 - SC-22760 - WB: 1/1000 - IF: 1/1000
Mouse monoclonal anti-PML - Santa Cruz - PG-M3 - SC-966 - WB: N.A. - IF: 1/1000
Mouse monoclonal anti-H3 - Cell Signaling - 1B1B2 - #14269S - WB: 1/20000 - IF: N.A.
Mouse monoclonal anti-RNA polymerase II - Millipore - 8WG16 - #05-952 - WB: 1/1000 - IF: N.A.
Mouse monoclonal anti-SUMO1 21C7 - DSHB - 21C7 - SUMO1 21C7 - WB: 1/250 - IF: N.A.
Mouse monoclonal anti-SUMO1 76-86 - DSHB - - SUMO1 76-86 - WB: 1/250 - IF: N.A.
Mouse monoclonal anti-SMUO-2 8A2 - DSHB - - SMUO-2 8A2 - WB: 1/250 - IF: N.A.
Mouse monoclonal anti-SUMO 6F2 - DSHB - - SUMO 6F2 - WB: 1/250 - IF: N.A.
Mouse monoclonal anti-caspase 3 - Santa Cruz - 31A1067 - SC-56053 - WB: 1/1000 - IF: N.A.
Mouse monoclonal anti-USP14 - Santa Cruz - 6E6 - SC-100630 - WB: 1/1000 - IF: N.A.
Rabbit monoclonal anti-S6 ribosomal protein - Cell Signaling - 5G10 - #2217S - WB: 1/1000 - IF: N.A.
Rabbit monoclonal anti-P-S6 ribosomal protein - Cell Signaling - D57,2,2E - #4858S - WB: 1/1000 - IF: N.A.
Rabbit polyclonal anti-P-4EBP1 - Cell Signaling - - #9459S - WB: 1/1000 - IF: N.A.
Rabbit monoclonal anti-4EBP1 - Cell Signaling - 53H11 - #9644S - WB: 1/1000 - IF: N.A.
Mouse monoclonal anti-MYC - This paper - - homemade - WB: 1/1000 - IF: N.A.
Mouse monoclonal anti-MCL-1 - Santa Cruz - B-6 - SC-74436 - WB: 1/500 - IF: N.A.
Mouse monoclonal anti-NOXA - Santa Cruz - 114C307 - SC-56169 - WB: 1/500 - IF: N.A.
Mouse monoclonal anti-PUMA - Santa Cruz - G-3 - SC-374223 - WB: 1/500 - IF: N.A.
Rabbit monoclonal anti-BAK - Cell Signaling - D2E11 - #5023T - WB: 1/1000 - IF: N.A.
Rabbit monoclonal anti-BAX - Cell Signaling - D4E4 - #12105T - WB: 1/1000 - IF: N.A.
Rabbit monoclonal anti-BIM - Cell Signaling - C34C5 - #2933T - WB: 1/1000 - IF: N.A.
Rabbit monoclonal anti-RAD23B - Cell Signaling - D4W7F - #13525 - WB: 1/1000 - IF: 1/1000
Mouse polyclonal anti-RPL29 - Abnova - B01P - H00006159-B01P - WB: N.A. - IF: 1/1000
Rabbit polyclonal anti-RPL15 - Proteintech - - 16740-1-AP - WB: N.A. - IF: 1/1000
Mouse monoclonal anti-Phospho-H2AX (ser139) - Millipore - JBW301 - 05-636-I - WB: 1/1000 - IF: N.A.
Mouse monoclonal anti-HSP70 - Santa Cruz - C92F3A-5 - SC-66048 - WB: 1/1000 - IF: N.A.
Mouse monoclonal anti-HIF1alpha - Santa Cruz - 28b - SC-13515 - WB: 1/1000 - IF: N.A.
Rabbit monoclonal Anti-Ubiquitin K48 - Abcam - EP8589 - ab140601 - WB: 1/1000 - IF: 1/1000
Mouse monoclonal anti-NPM1 - Thermofisher - FC-61991 - #32-5200 - WB: N.A. - IF: 1/1000
Goat polyclonal anti-Rabbit Alexa fluor 594 - Invitrogen - - A11012 - WB: 1/1000 - IF: N.A.
Goat polyclonal anti-Mouse Alexa fluor 594 - Invitrogen - - A11005 - WB: 1/1000 - IF: N.A.
Goat polyclonal anti-Rabbit Alexa fluor 488 - Invitrogen - - A11008 - WB: 1/1000 - IF: N.A.
Goat polyclonal anti-Mouse Alexa fluor 488 - Invitrogen - - A11029 - WB: 1/1000 - IF: N.A.
Goat polyclonal anti-Mouse HRP - Jackson Immunoresearch - - 115-036-003 - WB: N.A. - IF: 1/1000
Goat polyclonal anti-Rabbit HRP - Jackson Immunoresearch - - 111-036-003 - WB: N.A. - IF: 1/1000

Validation

We chose these antibodies based on information provided in product data sheets, literature and following our own experiments.
Mouse monoclonal anti-Fibrillarin - validation from the manufacturer's website https://www.scbt.com/fr/p/fibrillarin-antibody-b-1
Mouse monoclonal anti-ADRM1 - validation by western blotting using siRNA
Mouse monoclonal anti-FK2 - validation from the manufacturer's website https://www.merckmillipore.com/INTL/fr/product/Anti-Ubiquitinylated-proteins-Antibody-clone-FK2,MM_NF-04-263
Mouse monoclonal anti-LDH - validation from the manufacturer's website https://www.scbt.com/p/ldh-antibody-h-10
Mouse monoclonal anti-PA28γ (PSME3) - validation by western blotting using siRNA
Mouse monoclonal anti-SC35 - validation from the manufacturer's website https://www.scbt.com/p/p-sc35-antibody-sc-35
Mouse monoclonal anti-PSMB1 - validation from the manufacturer's website https://www.scbt.com/p/20s-proteasome-beta1-antibody-d-9?requestFrom=search
Mouse monoclonal anti-PSMB2 - validation from the manufacturer's website https://www.scbt.com/p/20s-proteasome-beta7-antibody-h-3?requestFrom=search
Mouse monoclonal anti-PSMB4 - validation from the manufacturer's website https://www.scbt.com/fr/p/psmb4-antibody-h-3
Rabbit polyclonal anti-PSMB5 - validation by western blotting using siRNA
Rabbit monoclonal anti-PSMB6 - validation by western blotting using siRNA
Rabbit monoclonal anti-PSMB7 - validation by western blotting using siRNA
Rabbit polyclonal anti-PSMD11 - validation by western blotting using siRNA
Rabbit monoclonal anti-PSMD14 - validation by western blotting using siRNA
Mouse monoclonal anti-PSMD4 - validation by western blotting using siRNA
Rabbit polyclonal anti-UCH37 - validation by western blotting using siRNA
Rabbit polyclonal anti-PSMD7 - validation by western blotting using siRNA
Mouse monoclonal anti-RAD23B - validation by western blotting using siRNA
Mouse monoclonal anti-Tubulin - validation from the manufacturer's website https://www.scbt.com/fr/p/alpha-tubulin-antibody-b-5-1-2
Mouse monoclonal anti-Ub - validation from the manufacturer's website https://datasheets.scbt.com/sc-8017.pdf
Rabbit polyclonal anti-c-fos - validation from the manufacturer's website https://www.scbt.com/fr/p/c-fos-antibody-h-125
Mouse monoclonal anti-PARP1 - validation from the manufacturer's website https://www.scbt.com/fr/p/parp-1-antibody-f-2
Mouse monoclonal anti-p53 - validation by western blotting using siRNA
Mouse monoclonal anti-c-jun - validation from the manufacturer's website https://www.scbt.com/fr/p/c-jun-antibody-g-4
Rabbit polyclonal anti-53BP1 - validation from the manufacturer's website https://www.scbt.com/fr/p/53bp1-antibody-h-300
Mouse monoclonal anti-PML - validation from the manufacturer's website https://www.scbt.com/fr/p/pml-antibody-pg-m3
Mouse monoclonal anti-H3 - validation from the manufacturer's website https://www.cellsignal.com/products/primary-antibodies/histone-h3-1b1b2-mouse-mab/14269
Mouse monoclonal anti-RNA polymerase II - validation from the manufacturer's website https://www.emdmillipore.com/CA/en/product/Anti-RNA-Polymerase-II-Antibody-CTD-Antibody-clone-8WG16,MM_NF-05-952-I-100UG?ReferrerURL=https%3A%2F%2Fwww.google.com%2F&bd=1
Mouse monoclonal anti-SUMO1 21C7 - validation from the manufacturer's website https://dshb.biology.uiowa.edu/

SUMO-1-21C7
Mouse monoclonal anti-SUMO1 76-86 - validation from the manufacturer's website https://dshb.biology.uiowa.edu/SUMO1-76-86
Mouse monoclonal anti-SMUO-2 8A2 - validation from the manufacturer's website https://dshb.biology.uiowa.edu/SUMO-2-8A2
Mouse monoclonal anti-SUMO 6F2 - validation from the manufacturer's website https://dshb.biology.uiowa.edu/SUMO-6F2
Mouse monoclonal anti-caspase 3 - validation from the manufacturer's website https://www.scbt.com/fr/p/caspase-3-antibody-31a1067
Mouse monoclonal anti-USP14 - validation by western blotting using siRNA
Rabbit monoclonal anti-S6 ribosomal protein - validation from the manufacturer's website https://www.cellsignal.com/products/primary-antibodies/s6-ribosomal-protein-5g10-rabbit-mab/2217
Rabbit monoclonal anti-P-S6 ribosomal protein - validation from the manufacturer's website https://www.cellsignal.com/products/primary-antibodies/phospho-s6-ribosomal-protein-ser235-236-d57-2-2e-xp-rabbit-mab/4858?site-search-type=Products&N=4294956287&Ntt=%234858s&fromPage=plp&_requestid=111621
Rabbit polyclonal anti-P-4EBP1 - validation from the manufacturer's website https://www.cellsignal.com/products/primary-antibodies/phospho-4e-bp1-thr37-46-antibody/9459?site-search-type=Products&N=4294956287&Ntt=%239459s&fromPage=plp&_requestid=111634
Rabbit monoclonal anti-4EBP1 - validation from the manufacturer's website https://www.cellsignal.com/products/primary-antibodies/4e-bp1-53h11-rabbit-mab/9644?site-search-type=Products&N=4294956287&Ntt=%239644s&fromPage=plp&_requestid=111652
Mouse monoclonal anti-MYC - validation from the manufacturer's website
Mouse monoclonal anti-MCL-1 - validation from the manufacturer's website https://www.scbt.com/p/mcl-1-antibody-b-6
Mouse monoclonal anti-NOXA - validation by western blotting using siRNA
Mouse monoclonal anti-PUMA - validation by western blotting using siRNA
Rabbit monoclonal anti-BAK - validation from the manufacturer's website https://www.cellsignal.com/products/primary-antibodies/bax-d2e11-rabbit-mab/5023
Rabbit monoclonal anti-BAX - validation from the manufacturer's website https://www.cellsignal.com/products/primary-antibodies/bak-d4e4-rabbit-mab/12105?site-search-type=Products&N=4294956287&Ntt=%2312105t&fromPage=plp&_requestid=111718
Rabbit monoclonal anti-BIM - validation from the manufacturer's website https://www.cellsignal.com/products/primary-antibodies/bim-c34c5-rabbit-mab/2933?site-search-type=Products&N=4294956287&Ntt=%232933t&fromPage=plp&_requestid=111731
Rabbit monoclonal anti-RAD23B - validation by western blotting using siRNA
Mouse polyclonal anti-RPL29 - validation from the manufacturer's website http://www.abnova.com/products/products_detail.asp?catalog_id=H00006159-B01P
Rabbit polyclonal anti-RPL15 - validation from the manufacturer's website https://www.ptglab.com/products/RPL15-Antibody-16740-1-AP.htm
Mouse monoclonal anti-Phospho-H2AX (ser139) - validation from the manufacturer's website https://www.emdmillipore.com/CA/en/product/Anti-phospho-Histone-H2A.X-Ser139-Antibody-clone-JBW301,MM_NF-05-636-I?ReferrerURL=https%3A%2F%2Fwww.google.com%2F&bd=1
Mouse monoclonal anti-HSP70 - validation from the manufacturer's website https://www.scbt.com/fr/p/hsp-70-antibody-c92f3a-5and heat shock stress by western blotting
Mouse monoclonal anti-HIF1alpha - validation from the manufacturer's website https://www.scbt.com/p/hif-1alpha-antibody-28b?requestFrom=search and by hypoxia inducton on cells
Rabbit monoclonal Anti-Ubiquitin K48 - validation from the manufacturer's website https://www.abcam.com/ubiquitin-linkage-specific-k48-antibody-ep8589-ab140601.html
Mouse monoclonal anti-NPM1 - validation from the manufacturer's website https://www.thermofisher.com/antibody/product/NPM1-Antibody-clone-FC-61991-Monoclonal/32-5200 and nuclear localization by immonufluorescence
Goat polyclonal anti-Rabbit Alexa fluor 594 - validation from the manufacturer's website https://www.thermofisher.com/antibody/product/Goat-anti-Rabbit-IgG-H-L-Cross-Adsorbed-Secondary-Antibody-Polyclonal/A-11012
Goat polyclonal anti-Mouse Alexa fluor 594 - validation from the manufacturer's website https://www.thermofisher.com/antibody/product/Goat-anti-Mouse-IgG-H-L-Cross-Adsorbed-Secondary-Antibody-Polyclonal/A-11005
Goat polyclonal anti-Rabbit Alexa fluor 488 - validation from the manufacturer's website https://www.thermofisher.com/antibody/product/Goat-anti-Rabbit-IgG-H-L-Cross-Adsorbed-Secondary-Antibody-Polyclonal/A-11008
Goat polyclonal anti-Mouse Alexa fluor 488 - validation from the manufacturer's website https://www.thermofisher.com/antibody/product/Goat-anti-Mouse-IgG-H-L-Highly-Cross-Adsorbed-Secondary-Antibody-Polyclonal/A-11029
Goat polyclonal anti-Mouse HRP - validation from the manufacturer's website https://www.jacksonimmuno.com/catalog/products/115-036-003
Goat polyclonal anti-Rabbit HRP - validation from the manufacturer's website https://www.jacksonimmuno.com/catalog/products/111-036-003

Eukaryotic cell lines

Policy information about cell lines

Cell line source(s)

HEK293T (ATCC, CRL-3216)
IMR90 (ATCC, CCL-186)
3T3-L1 (ATCC, CL-173)
HDLF (gift from Dr Eliot Drobetsky)
MCF7 (gift from Dr Sylvie Mader)
NCI-H2199 (gift from Dr Wei Gu)
AT3 (gift from Rangnekar Vivek)
RAW264.7 (ATCC, SC-6003)
HCT116 (ATCC, CCL-247)

	C2C12 (ATCC, CRL-1772)<br>PC-3 (ATCC, CRL-1435)<br>Mia-PaCa-2 (ATCC, CRL-1420)<br>T47D (ATCC, HTB-133)<br>LLC (gift from Dr Bruno Larrivée)<br>MDA-MB-231<br>HeLa (gift from Dr Eliot Drobetsky)<br>NIH3T3 (ATCC, CRL-1658)<br>Cos-7 (gift from Dr Eric Milot)<br>mESCs (gift from Dr Eric Milot)<br>HUVEC (gift from Dr Bruno Larrivée)
Authentication	HEK293, IMR90, 3T3L1, RAW264.4, HCT116, C2C12, NIH3T3, T47D, Mia-PaCa2, PC-3 cells were obtained from ATCC and several aliquots were frozen to avoid extended cell culture. The cells were discarded after few weeks of cell culture.<br>The rest of the cell lines were not authenticated. Cells were carefully monitored for typical morphology, growth capacity and other caracteristics such as cell senescence and differentiation as well as expression of key proteins.
Mycoplasma contamination	All cell lines are tested negative for mycoplasma contamination.
Commonly misidentified lines<br>(See ICLAC register)	No misidentified cell lines were used in this study.

Palaeontology

Specimen provenance	Not applicable
Specimen deposition	Not applicable
Dating methods	Not applicable

☐ Tick this box to confirm that the raw and calibrated dates are available in the paper or in Supplementary Information.

Animals and other organisms

Policy information about studies involving animals; ARRIVE guidelines recommended for reporting animal research

Laboratory animals	Athymic nude NU/J mice (Foxn1nu; The Jackson Laboratory, #002019), bith sexes used, 6 weeks old. Mice were maintained in large group houses of 4-5 members, in airfiltered cages with controlled temperature (20 °C) and humidity (50%), in a 12 h light/dark cycle, and were given access to food and water ad libitum.
Wild animals	No wild animal were used for this study.
Field-collected samples	No field collected samples were used.
Ethics oversight	Animal Care Committee of the research center of the Maisonneuve Rosemont hospital in agreement with the guidelines established by the Canadian Council on Animal Care.

Note that full information on the approval of the study protocol must also be provided in the manuscript.

Human research participants

Policy information about studies involving human research participants

Population characteristics	Not applicable
Recruitment	Not applicable
Ethics oversight	Not applicable

Note that full information on the approval of the study protocol must also be provided in the manuscript.

Clinical data

Policy information about clinical studies

All manuscripts should comply with the ICMJE guidelines for publication of clinical research and a completed CONSORT checklist must be included with all submissions.

Clinical trial registration	Not applicable
Study protocol	Not applicable
Data collection	Not applicable

Outcomes	Not applicable

ChIP-seq

Data deposition

☐ Confirm that both raw and final processed data have been deposited in a public database such as GEO.

☐ Confirm that you have deposited or provided access to graph files (e.g. BED files) for the called peaks.

Data access links<br>*May remain private before publication.*	Not applicable
Files in database submission	Not applicable
Genome browser session<br>(e.g. UCSC)	Not applicable

Methodology

Replicates	Not applicable
Sequencing depth	Not applicable
Antibodies	Not applicable
Peak calling parameters	Not applicable
Data quality	Not applicable
Software	Not applicable

Flow Cytometry

Plots

Confirm that:

☒ The axis labels state the marker and fluorochrome used (e.g. CD4-FITC).

☒ The axis scales are clearly visible. Include numbers along axes only for bottom left plot of group (a 'group' is an analysis of identical markers).

☒ All plots are contour plots with outliers or pseudocolor plots.

☒ A numerical value for number of cells or percentage (with statistics) is provided.

Methodology

Sample preparation	Cells were washed with PBS and harvested by trypsinization. Cells were centrifugated, washed once again with PBS and fixed with 75 % cold ethanol. Cells were centrifugated and resuspended in PBS containing 100 µg/ml RNase A and incubated at 37°C for 30 min. To stain DNA, propidium iodide was added at 50µg/ml final concentration.
Instrument	Cell cycle experiments were performed on a FACSCalibur flow cytometer with CellQuestPro software (BD Biosciences).
Software	Acquisition was performed on CellQuestPro software; data were analysed with FlowJo v10.6.1
Cell population abundance	10.000 cells from the gating population were acquired by condition.
Gating strategy	Populations were first gate according to FSC/SSC. Then, we gated a single cell population with FL2-A/FL2-W excluding cell debris and doublets. Histograms correspond to cell count/FL2-A.

☒ Tick this box to confirm that a figure exemplifying the gating strategy is provided in the Supplementary Information.

Magnetic resonance imaging

Experimental design

Design type	Not applicable
Design specifications	Not applicable

Behavioral performance measures	Not applicable

Acquisition

Imaging type(s)	Not applicable
Field strength	Not applicable
Sequence & imaging parameters	Not applicable
Area of acquisition	Not applicable

Diffusion MRI ☐ Used ☐ Not used

Preprocessing

Preprocessing software	Not applicable
Normalization	Not applicable
Normalization template	Not applicable
Noise and artifact removal	Not applicable
Volume censoring	Not applicable

Statistical modeling & inference

Model type and settings	Not applicable
Effect(s) tested	Not applicable

Specify type of analysis: ☐ Whole brain ☐ ROI-based ☐ Both

Statistic type for inference (See Eklund et al. 2016)	Not applicable
Correction	Not applicable

Models & analysis

n/a	Involved in the study
☐	☐ Functional and/or effective connectivity
☐	☐ Graph analysis
☐	☐ Multivariate modeling or predictive analysis

Functional and/or effective connectivity	Not applicable
Graph analysis	Not applicable
Multivariate modeling and predictive analysis	Not applicable
